# Growing and Flowering in a Changing Climate: Effects of Higher Temperatures and Drought Stress on the Bee-Pollinated Species *Impatiens glandulifera* Royle

**DOI:** 10.3390/plants10050988

**Published:** 2021-05-15

**Authors:** Charlotte Descamps, Najet Boubnan, Anne-Laure Jacquemart, Muriel Quinet

**Affiliations:** Earth and Life Institute—Agronomy, UCLouvain, Croix du Sud 2, box L7.05.14, 1348 Louvain-la-Neuve, Belgium; najet.boubnan@hotmail.com (N.B.); anne-laure.jacquemart@uclouvain.be (A.-L.J.); muriel.quinet@uclouvain.be (M.Q.)

**Keywords:** plant–pollinator interactions, water stress, drought, heat, abiotic stress, floral signals, nectar, pollen, floral rewards, bee-pollinated species

## Abstract

Drought and higher temperatures caused by climate change are common stress conditions affecting plant growth and development. The reproductive phase is particularly sensitive to stress, but plants also need to allocate their limited resources to produce floral traits and resources to attract pollinators. We investigated the physiological and floral consequences of abiotic stress during the flowering period of *Impatiens glandulifera*, a bee-pollinated species. Plants were exposed to three temperatures (21, 24, 27 °C) and two watering regimes (well-watered, water stress) for 3 weeks. Not all parameters measured responded in the same manner to drought and/or heat stress. Drought stress induced leaf senescence, decreasing leaf number by 15–30% depending on growth temperature. Drought also reduced photosynthetic output, while temperature rise affected stomatal conductance. The number of flowers produced dropped 40–90% in response to drought stress, while higher temperatures shortened flower life span. Both stresses affected floral traits, but flower resources diminished in response to higher temperatures, with lower nectar volume and pollen protein content. We conclude that increased temperatures and drought stress, which are becoming more frequent with climate change, can negatively affect flowering, even if plants deploy physiological resistance strategies.

## 1. Introduction

Climate change is responsible for more frequent and unpredictable variation in daily and seasonal temperatures, as well as drought [1]. These consequences of a warming climate are detrimental to plants, especially when they co-occur [1,2,3]. In temperate regions, temperature rise and drought stresses are more likely to affect plants simultaneously during the Spring and Summer seasons, which are crucial periods for plant–pollinator interactions. Entomophilous plants attract pollinators with their floral traits and resources (nectar and pollen) to ensure pollination [4,5]. Bees rely exclusively on these floral resources as their food sources. The plant reproductive stage is particularly sensitive to higher temperatures and drought [6], which may severely disrupt plant–pollinator interactions by negatively affecting floral signals and resources [7,8].

Plants suffer from heat stress when temperatures exceed their optimal growing temperature, a condition often accompanied by drought stress [6,9]. These stressful conditions may impair vital physiological processes such as stomatal conductance, photosynthesis and respiration [2,10,11]. Co-occurrence of these two abiotic stresses initiates complex plant responses that cannot be deduced from single stress responses [12,13]. For example, heat and drought have conflicting effects on stomatal conductance. While plants close their stomata when faced with water stress, they open them at a higher temperature to maintain transpiration and leaf cooling [14]. In addition, closed stomata reduce CO_2_ uptake and, thus, photosynthetic output [2,15]; photosystem II is susceptible to a rise in temperature, leading to lower photosynthesis during heat stress [16]. Growing in such challenging environments limits resources available for plant reproduction [17]. Although heat and drought stress negatively affect all plant developmental stages, the flowering period is particularly sensitive to abiotic stresses [18,19,20], which can result in fewer flowers, higher flower abortion rates and impaired flower development and fertility, leading to lower reproductive success [6,19]. 

During the flowering period, entomophilous plant species attract pollinators with floral traits (floral display, scent, color) and resources such as nectar and pollen [21]. These resources are crucial to bees, constituting their sole food sources. Nectar is a major sugar source, while pollen provides amino acids, proteins and lipids [22,23,24]. The volume and quality of these resources will contribute to flower attractiveness, as a high sugar content in nectar and a high polypeptide content in pollen are more attractive to pollinators compared to resources of lower quality or quantity [25,26,27,28,29], as well as bee development and survival [30,31,32]. The modifications of floral traits and floral resources stemming from higher temperatures and drought may, therefore, have dire consequences on plant–pollinator interactions. However, how water stress, rising temperatures and their combination affect floral traits and resources in entomophilous species has not been extensively investigated to date [7,33,34,35].

This study aimed to investigate the effects of higher temperatures and drought, individually and in combination, on the reproductive phase of the bee-pollinated species Himalayan balsam (*Impatiens glandulifera*, Balsaminaceae). *Impatiens glandulifera* was introduced from the Himalayas for horticulture purposes and is now one of the most invasive annuals in Europe [36]. It grows in areas with medium shade and requires soil moisture; it can grow on nutrient-rich to -poor soil [36]. We selected this plant as a model for its high floral resource production. Flowers of *I. glandulifera* are highly attractive to pollinators and produce large amounts of nectar and pollen (1.3 × 10^6^ pollen grains and 7.3 µL of nectar per flower [36]). Although *I. glandulifera* could be considered as self-compatible, it shows low autonomous selfing and bees visit flowers up to 250 times during the flower life span [36]. Our hypothesis was that concomitant heat and drought stress would affect plant growth and physiology, decreasing the volume and quality of floral resources and modifying floral traits. We addressed the following questions: (1) Do concomitant temperature and water stresses affect plant growth, plant physiology, floral traits and resources? (2) Do these stresses influence floral traits and floral resources directly or indirectly by modulating plant growth and physiology?

## 2. Results

Plants were grown over their flowering period under two watering conditions (well-watered, WW, and water-stressed, WS) and three temperature regimes (21, 24, 27 °C), resulting in six treatments (21WW, 21WS, 24WW, 24WS, 27WW, 27WS). 

### 2.1. Vegetative and Physiological Parameters

**Figure 1:**
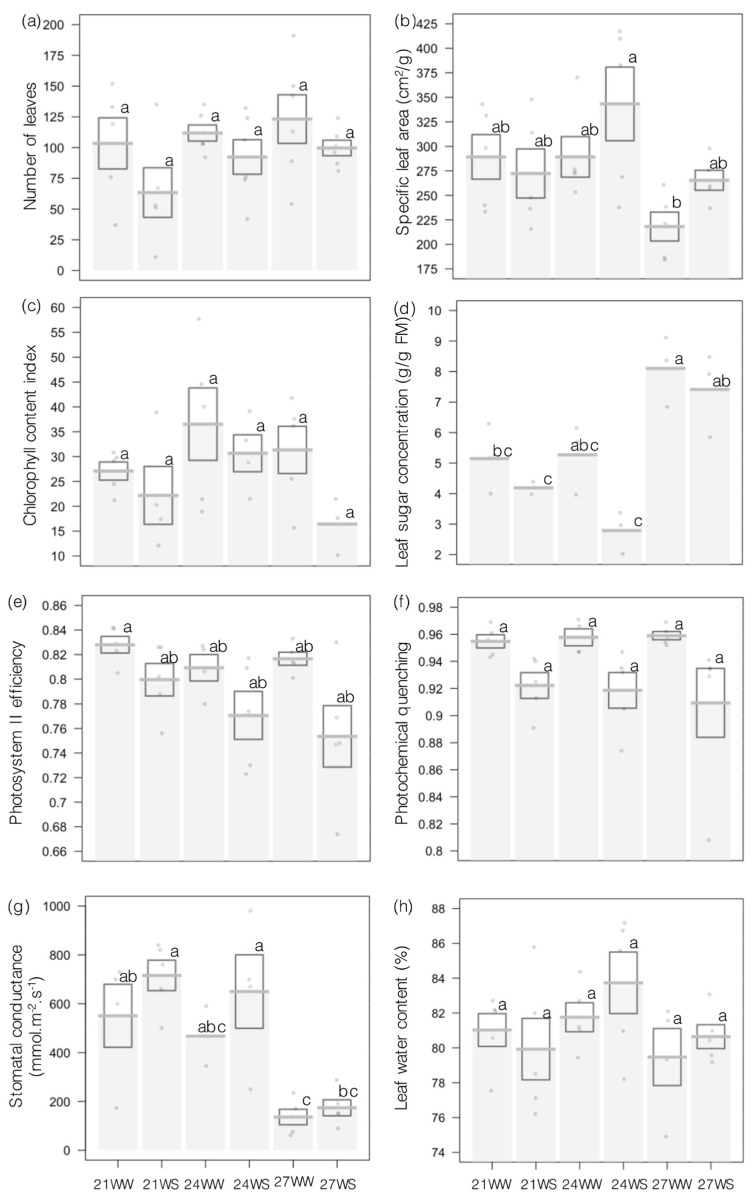
Effects of increasing temperatures and water stress on vegetative and physiological parameters of *Impatiens glandulifera* plants 2 weeks after beginning treatment. (**a**) Number of leaves, (**b**) specific leaf area, (**c**) chlorophyll content index, (**d**) leaf sugar concentration, (**e**) photosystem II efficiency, (**f**) photochemical quenching, (**g**) stomatal conductance and (**h**) leaf water content. N = 5 per treatment, except for leaf sugar content, with N = 3. Data are presented as means ± standard errors (SE) as barplots, with individual data points shown in gray. Treatments followed by different letters are significantly different at *p* < 0.05 21, 21 °C; 24, 24 °C; 27, 27 °C; WS, water-stressed; WW, well-watered. 21WW = control.

Drought stress decreased leaf number, while higher temperatures had no significant effect (Figure 1a, Table 1). Specific leaf area (SLA) varied with temperature, but not with soil water content (Figure 1b, Table 1), as plants grown at 21 and 24 °C had a higher mean SLA than plants grown at 27 °C. 

We then determined the effects of both stresses on photosynthesis: chlorophyll content was not significantly affected by drought or rising temperatures (Figure 1c, Table 1). However, drought stress negatively affected the light phase of photosynthesis by lowering photosystem II efficiency and photochemical quenching (Figure 1e,f, Table 1). Soluble sugar content in leaves varied with both temperature and soil water moisture: leaves from plants grown at 27 °C accumulated more sugar compared to those grown at 21 or 24 °C, and sugar content decreased in plants subjected to drought, mainly at 24 °C (Figure 1d, Table 1). Finally, we assessed the influence of temperature and drought on gas exchange: stomatal conductance decreased at 27 °C compared to 21 and 24 °C but was not affected by drought (Figure 1g, Table 1). Leaf water content remained around 80% under all growth conditions (Figure 1h, Table 1).

### 2.2. Floral Traits and Resources

**Figure 2:**
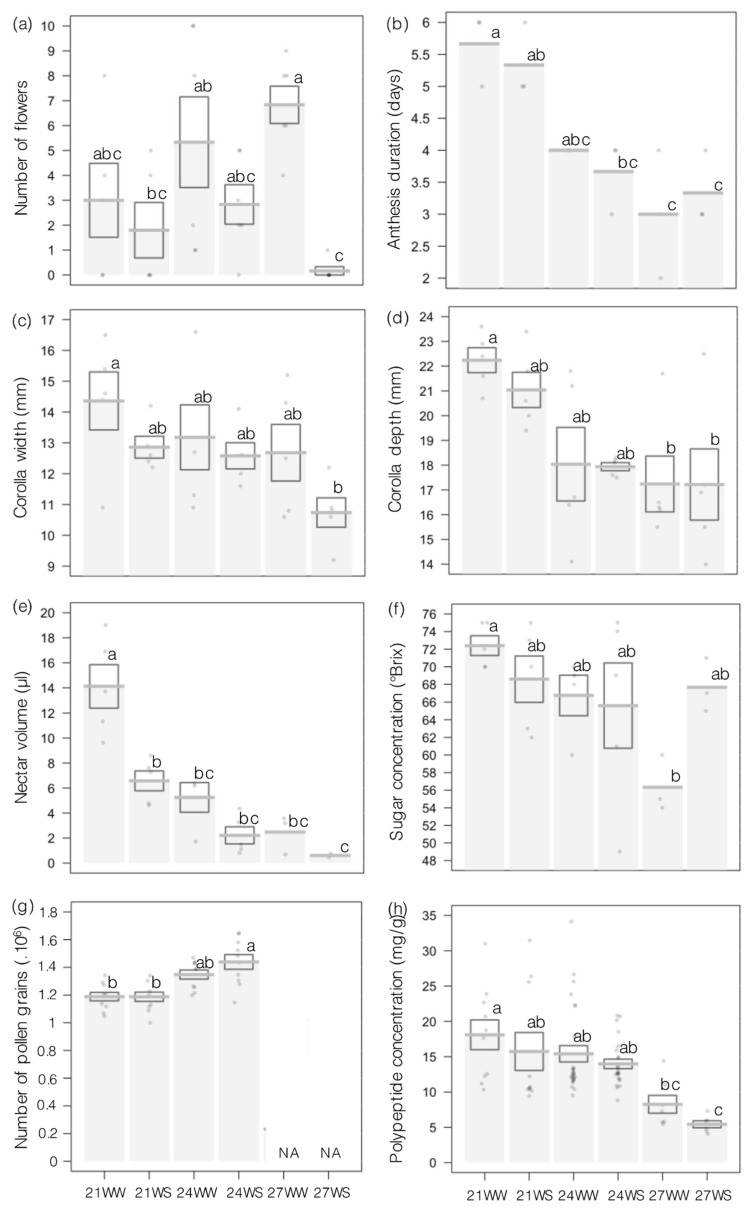
Effects of increasing temperature and water stress on floral traits and resources of *Impatiens glandulifera* plants 2 weeks after beginning treatment. (**a**) Number of flowers per plant, (**b**) anthesis duration per flower, (**c**) corolla width, (**d**) corolla depth, (**e**) nectar volume per flower, (**f**) sugar concentration in nectar, (**g**) number of pollen grains per flower and (**h**) polypeptide concentration in pollen. N = 5 per treatment, except for number of pollen grains and polypeptide content, with N = 10. Data are presented as means ± standard errors (SE) as barplots with individual data points shown in gray. Treatments followed by different letters are significantly different at *p* < 0.05. 21, 21 °C; 24, 24 °C; 27, 27 °C; WS, water-stressed; WW, well-watered. 21WW = control.

Drought stress resulted in fewer flowers being produced, especially at 27 °C (Figure 2a, Table 1); plants exposed to the 27WS treatment had almost no flowers after the 2-week treatment. Furthermore, higher temperatures shortened the duration of flower anthesis, while soil water moisture did not contribute significantly (Figure 1b, Table 1). Flowers remained open for only 3 days when subjected to 27 °C, but a full 5–6 days when grown at 21 and 24 °C (Figure 2b, Table 1). Drought stress modified flower shape by diminishing corolla width by 25% at 27WS compared to 21WW (Figure 2c, Table 1). Higher temperatures reduced corolla depth by 15% at 24 and 27 °C relative to growth at 21 °C, under both well-watered and drought conditions (Figure 2d, Table 1).

The volume and quality of floral resources produced were mainly affected by the rise in temperature (Table 1). Nectar volume dropped drastically in response to both drought stress and higher temperatures, reaching 14 µL per flower grown at 21WW but only 0.5 µL per flower grown at 27WS (Figure 2e, Table 1). By contrast, the sugar concentration of nectar remained fairly constant and decreased markedly only in 27WW flowers to 56 °Brix relative to 72 °Brix in 21WW flowers (Figure 2f, Table 1). Higher temperatures and drought stress lowered the sugar content in nectar per flower from 14.1 ± 1.8 mg at 21WW to 0.6 ± 0.1 mg at 27WS. Another measure of the quantity of floral resources, the number of pollen grains per flower, increased when grown at 24 °C compared to 21 °C under both well-watered and drought conditions (Figure 2g, Table 1). Many anthers were empty when plants were grown at 27 °C, preventing us from scoring pollen grains under these conditions. Instead, we used these precious and limited pollen samples collected at 27 °C to determine polypeptide content, another proxy of pollen quality. We observed that polypeptide content was negatively affected by higher temperatures but not by drought (Figure 2 h, Table 1), with polypeptide content decreasing by 65% between the 21WW and 27WS treatments.

### 2.3. Principal Component Analysis

**Figure 3:**
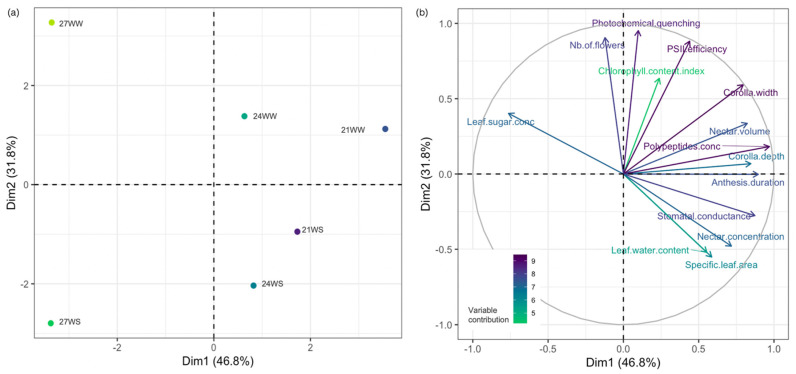
Principal component analysis (PCA) of vegetative, physiological and floral traits and resources of *Impatiens glandulifera* plants grown under three temperatures (21, 24, 27 °C) and two watering regimes (WW, well-watered; WS, water stressed) for 2 weeks. (**a**) Individual graph showing the different treatments; (**b**) variable graph of PCA showing vegetative, physiological, floral traits and floral reward parameters. conc = concentration, PSII = photosystem II. 21, 21 °C; 24, 24 °C; 27, 27 °C; WS, water-stressed; WW, well-watered. 21WW = control.

We performed a principal component analysis of all phenotypic values collected in this study. The first two principal components explained 78.6% of the observed variance (Figure 3). Axis 1 largely separated samples by their temperature treatments (21, 24 vs. 27 °C, Figure 3a) and as a function of their underlying physiology (stomatal conductance, leaf sugar content) and floral resources (polypeptide content) (Figure 3b). Axis 2 discriminated samples based on their water status, with a strong contribution from the number of flowers produced per plant and photochemical quenching values. In particular, plants grown in the 27WS and 27WW treatments clustered away from all other treatments, highlighting the high level of stress associated with these growth conditions.

## 3. Discussion

Higher temperatures and drought stress affected physiological traits as well as floral traits and resources in the entomophilous species *I. glandulifera.* However, not all parameters responded in the same direction to a rise in temperature or to drought, alone or in combination. Drought stress affected leaf number and the light phase of photosynthesis, while higher temperatures targeted leaf size and stomatal conductance. We observed distinct effects of each stress on the reproductive phase, as drought stress reduced the number of flowers produced, while higher temperatures shortened flower life span. Both stresses influenced floral traits; floral resources were themselves mainly affected by higher temperatures. Plant growth and development can be differently affected by heat and drought stresses [6,37], and plants exhibiting resistance to one stress are not always resistant to other stresses or to their combination [10,38,39], highlighting the importance of considering combinations of stresses when attempting to predict plant responses to climate change. Our results demonstrated that plants exposed to both high temperatures of 27 °C and drought stress (27WS) were more stressed relative to those exposed to all other growth conditions tested here.

Drought stress reduced the number of leaves by 15–30% depending on the growth temperature, a common consequence of water stress observed in multiple plant species [9,40,41,42]. Leaf senescence is induced by the phytohormone ethylene, whose biosynthesis is stimulated by drought [43]. By reducing the number of leaves, the plant decreases its transpiration surface and so its need for water [44]. However, this strategy also leads to a lower total photosynthetic output, resulting in decreased carbohydrate production, which affects yield for many crop species [45,46]. Furthermore, we observed that water stress negatively affected the light phase of photosynthesis. Although chlorophyll content remained constant across all treatments, photosystem II efficiency and photochemical quenching were both reduced in plants experiencing drought. As a result, some of the energy captured by the photosystems was dissipated without being used for photosynthesis, and a great number of the reaction centers of the photosystems were closed under water stress, making them unavailable to capture light [47]. Impairing the photosynthetic apparatus is a major effect of drought [48] that leads to diminished photosynthesis and carbohydrate production. Faced with drought stress, most plants close their stomata to decrease leaf transpiration [46,49,50]. We noticed no effect on stomatal conductance in response to drought in our experimental conditions, however. Stomatal responses vary greatly between plant species [51]. For example, drought did not affect stomatal conductance in other species such as Tartary buckwheat (*Fagopyrum tataricum*) and starflower (*Borago officinalis*) [12,52]. Leaf water content remained constant between well-watered and drought-stressed plants and specific leaf area was neither affected by water stress. This suggests either that I. *glandulifera* has developed mechanisms to limit water loss or that the stress was not severe to the point of reducing these parameters. Moreover, it cannot be excluded that the constant high relative humidity (80%) in the growth room limited the water stress at the leaf level and partly explained the stability of leaf water content, leaf area and stomatal conductance. In accordance with the decrease in photosynthesis, we observed a lower concentration of soluble sugars in the leaves in response to water stress. By decreasing photosynthetic rate and sugar production, water stress limited the resources available for flower development and plant reproduction.

*I. glandulifera* plants were relatively tolerant to higher temperatures during their vegetative phase, as only physiological parameters such as SLA and stomatal conductance were lower at 27 °C. As higher temperatures did not affect leaf water content, reduced SLA is most likely attributable to a reduction in leaf size, which has been reported under similar conditions in other plant species [52]. In agreement, cell elongation becomes impaired when temperatures exceed the optimum for plant growth [53]. In contrast to drought stress, the light phase of photosynthesis was not affected by higher temperatures in our study, although other studies showed that chlorophyll biosynthesis and light phase of photosynthesis are sensitive to heat in several plant species [46]. Such a discrepancy might be explained by the fact that a temperature of 27 °C may induce only a moderate stress in *I. glandulifera.* Similarly, Zhou et al. [13] reported that drought stress was a more potent inhibitor of the light-dependent phase of photosynthesis than higher temperatures in tomato (*Solanum lycopersicum*). However, our results showed that gas exchange, which modulates photosynthetic capacity, responded to higher temperatures in *I. glandulifera*. Stomatal conductance decreased at 27 °C, suggesting that the plants closed their stomata. By closing their stomata, plants reduce CO_2_ uptake, which can lead to a decrease in sugar synthesis by affecting the light independent phase of photosynthesis [54,55]. On the contrary, total soluble sugar concentrations increased in leaves grown at 27 °C compared to those grown at 21 or 24 °C but might reflect the smaller size of leaves grown at 27 °C. A similar response in the leaf concentration of soluble sugars has been noted in response to abiotic stress in other plant species [56]. Soluble sugars may also be used as osmolytes to maintain cell turgor by forming a concentration gradient to counteract water loss from the higher temperatures [57,58,59]. In response to abiotic stress, carbohydrates might become mobilized to mount cost-effective resistance mechanisms to the detriment of plant growth [59]. Limited sucrose export to sink organs will ultimately limit reproductive development [17,48]. Consistent with this notion, Ghanem et al. [56] indeed observed that although the concentration of soluble sugars was reported to increase in tomato leaves in response to abiotic stress, it decreased in inflorescences. They explained the failure of inflorescence development due to altered source–sink relationships [56].

In addition to affecting plant physiology and leaf sugar concentrations, drought stress and higher temperatures also influenced flower production, floral traits and floral resources in *I. glandulifera*. We observed damage to reproductive organs at 24 °C, indicating that the optimum temperature for reproductive growth is lower than for vegetative growth, as previously reported in several species [12,19]. The reproductive phase is typically more sensitive to abiotic stress than the vegetative phase of plant development [6,19]. Drought stress reduced the number of flowers per plant by 50% at 21 and 24 °C and by close to 100% at 27 °C, in agreement with previous reports [35,60,61]. By contrast, flower production remained unaffected by higher temperatures, although heat stress has been reported to reduce the number of flowers in several species [62,63]. However, higher temperatures did shorten the life span of flowers, an effect that was not observed in the context of drought stress. A shorter anthesis period may reduce the reproductive success of the flower. In addition, both drought stress and heat stress modified flower size, with the temperature reducing corolla depth and drought stress making the corolla narrower. Corolla size shows high plasticity in response to abiotic stress for annual species [64]. Maintaining large flowers with a long life span comes at a cost for plants [65,66]. One possible explanation for the negative influence of abiotic stresses on flower production and development may involve the investment of carbohydrates into resistance mechanisms at the expense of reproductive growth [17,56]. Floral organs continuously draw on resources to avoid reproductive failure under any growth condition, but perhaps even more so under abiotic stress when photosynthates might be in short supply [67,68,69]. The proper development of reproductive structures relies on the import of photoassimilates from leaves through the phloem [70]. When the capacity of reproductive organs to import and use assimilates is disturbed by abiotic stress, it leads to higher rates of fruit and seed abortion [48]. Moreover, abiotic stress may change the guild of pollinators attracted to flowers, as their corollas become smaller [71], and may even result in a morphological mismatch between flower size and that of the pollinator in severely stunted corollas [33,72]. The number of flowers per plant and their size largely contribute to the floral display plants use to attract pollinators [73,74]. A less conspicuous floral display may change pollinator behavior (e.g., by reducing the visitation rate or pollen deposition rate), with adverse consequences for plant reproduction [35].

Floral resources such as the amount of nectar produced were strongly reduced by higher temperatures and drought stress; many flowers grown at 27 °C had no nectar, although *I. glandulifera* is known for its high nectar production [36]. Abiotic stresses often significantly decrease nectar volume per flower [75,76]. Furthermore, the concentration of sugars in nectar was lower at 27 °C. Sugar concentration in nectar is typically less sensitive to abiotic stresses than nectar volume [35,75,77]; sugar concentration can remain unchanged in nectar even under stress [63,78]. However, annual species appear to be more sensitive to higher temperatures for floral resource production, as we previously observed a decrease in sugar concentration for salvation jane (*Echium plantagineum*) in a similar experiment [64]. Despite the higher sugar concentration measured in leaves at 27 °C, sugar content in fact decreased in nectar of the same plants. Again, soluble sugars may be used preferentially for vegetative organs rather than reproductive tissues, suggesting that assimilate partitioning between vegetative and reproductive organs is modified by abiotic stress [17]. Lower nectar production would also affect pollinator behavior. *Impatiens glandulifera* is highly attractive to bumblebees and honeybees due to its high nectar production [36], and nectar is the main source of sugar for these pollinators [22]. Less nectar production due to higher temperatures and drought stresses affect pollinator visitation rates and, thus, the reproductive success of the plant [63,76,79]. This could lead to affecting the invasiveness of *I. glandulifera,* since the invasive strategy of this species is based on high reproductive success [36,80].

*Impatiens glandulifera* also produce large amounts of pollen [36]. In our experiment, pollen production was higher at 24 °C than at 21 °C, while most anthers were empty at 27 °C. The number of pollen grains is determined very early during floral morphogenesis [81], and microsporogenesis is the most sensitive to abiotic stress [82,83,84]. Higher temperatures and drought stress reduce pollen quantity and viability [12], although viability was not investigated here. By contrast, protein concentration decreased in pollen with higher temperature, indicative of lower pollen quality. To the best of our knowledge, only one other recent study investigated the effect of higher temperatures on pollen quality but did not reveal a decrease in protein concentration [85]. Pollen protein content is a key parameter for both plant reproductive success and insect pollinator health. From the plant perspective, lower protein content in the pollen has been linked to a decrease in pollen viability [86]. Pollen grain maturation is a stress-sensitive process in many plant species [82,87]. Temperature rise reduces starch concentration in developing pollen grains, causing failures in their development [88,89]. This could be explained by the disruption in resource allocation to reproductive organs due to abiotic stresses, as previously mentioned. From the side of the pollinator, bees rely exclusively on floral resources for food and feed on pollen as their sole source of proteins, amino acids and sterols. The chemical composition of pollen can influence insect visitation behavior [28,30,86,90]. Furthermore, foraging pollen with low quality (i.e., reduced protein content) will affect the survival of larvae as well as adult survival and reproduction [31]. Thus, the production of little pollen of low quality due to higher temperatures and drought stress will directly affect both plant reproductive success and pollinator visits.

In conclusion, our results show that higher temperatures and drought stress negatively but differently influence physiological traits, floral traits and floral resources in the entomophilous species *I. glandulifera.* While plants attempt to adjust and maintain their physiological processes to support their survival in response to outside stressors, the resulting reallocation of resources will have negative consequences on reproductive organs. The combination of higher temperatures and drought stress was particularly deleterious during flowering. For entomophilous plants, this stage is critical, as they need to attract pollinating insects, some of which depend exclusively on their floral resources as food sources. The observed reduction in flower production, flower size and volume and quality of floral resources will directly affect plant–pollinator interactions, with negative consequences for both partners, although these aspects remain to be investigated.

## 4. Materials and Methods

### 4.1. Plants and Growth Condition

Himalayan balsam (*Impatiens glandulifera*) is an annual, entomophilous plant with large zygomorphic pink flowers that originated from the Himalayas and has spread worldwide as an ornamental. *Impatiens glandulifera* is one of the most invasive annual species in the world [91]. It has spread in the majority of temperate communities in Europe, growing in riparian biotopes and in other disturbed sites with good water and nutrient supply [36,80]. It is now considered as one of the 100 worst invasive species in Europe [36,80]. The stem stands erect and grows to 1–2 m in height, with lanceolate leaves. The lower sepal gradually contracts into a nectar spur. Stamens are fused by their anthers and form a brush that covers the stigma [92]. This species produces copious floral resources (~1.3 × 10^6^ pollen grains and 7.3 µL of nectar with 52% as sugars per flower [36]). *Impatiens glandulifera* flowers are, thus, very attractive to pollinators, each receiving up to 250 insect visits over their life span [36].

*Impatiens glandulifera* seedlings were collected from populations in Belgium (Court-Saint-Etienne, N 50°38′39′’ N 4°34′6′’ E and Jamioulx, 50°21′10′’ N, 4°24′45′’ E). Seedlings were transferred to 5 L pots containing universal peat compost (DCM, Amsterdam, Netherlands) and grown in the glasshouse at the University campus (Louvain-la-Neuve 50°39′58′′ N; 4°37′9′′ E, Belgium). Plants were watered daily with rainwater until the beginning of the experiment.

Plants received treatments at the beginning of their reproductive stage, when bolting occurs, flowering stems develop, and the first flowering buds become visible. Plants were exposed to three growth temperatures (21, 24 and 27 °C) and two watering regimes (well-watered, WW vs. water stress, WS) to investigate the effects of increasing temperature and water stress, individually and in combination, on plant growth and floral traits and resources. These conditions correspond to those encountered during the flowering period of the plant in Belgium and to temperatures expected in the context of climate change predictions. In total, 60 plants (10 plants per treatment) were subjected to one of six treatments: 21 °C well-watered (21WW), 21 °C water-stressed (21WS), 24 °C well-watered (24WW), 24 °C water-stressed (24WS), 27 °C well-watered (27WW) and 27 °C water-stressed (27WS); 21WW was considered as control condition based on the growing conditions encountered by *I. glandulifera* naturally in Belgium. Plants were transferred to growth chambers set to the following temperature cycles (day/night): 21 °C/19°C, 24 °C/22°C and 27 °C/25°C. Plants were grown in a long-day photoperiod (16 h light:8 h darkness), and relative humidity was maintained at 80 ± 10%. Illumination was provided by Philips HPIT 400 W lamps (Philips Lighting S.A., Brussels, Belgium), to a light intensity of 155 ± 20 μmol m^−2^ s^−1^ at canopy level (Skye Instruments Quantum Sensor quantum meter; Hansatech Instruments, Norfolk, UK). Each growth chamber housed the two watering regimes for each experimental temperature. The well-watered plants were watered daily (soil humidity ~50%), while the water-stressed plants were watered twice a week (soil humidity <30%). Water stress started after 1 week of acclimation to the growth chambers (this week was considered week 0). All measurements were taken 2 weeks after the beginning of water stress.

### 4.2. Vegetative and Physiological Parameters

The number of photosynthetically active leaves was assessed from five plants per treatment.

Physiological measurements were performed on the fifth youngest leaf (counted from the shoot apex) of five plants for each treatment, between 10 am and 3 pm. Chlorophyll fluorescence was monitored using a pulse-modulated fluorimeter (FMS II; Hansatech Instruments, Norfolk, UK). The collected parameters were photosystem II (PSII) efficiency (ΦPSII), which measures the proportion of light absorbed by PSII used in photochemistry, and qP, which indicates the proportion of PSII reaction centers that are open [47]. Leaf were dark-adapted for 30 min before illumination with a first pulse of 18,000 mmol m^−2^s^−1^ followed by constant illumination with actinic light (660 mmol m^−2^ s^−1^) for 2 min. The leaves were then exposed to a second saturating pulse of 18,000 mmol m^−2^ s^−1^. Chlorophyll content index (CCI) was measured using a chlorophyll meter (Opti-Sciences, CCM-200), with the measurement taken three times on the same leaf. An automatic porometer (AP4 System, Delta-T Devices) was used to measure conductance g_s_ on the abaxial leaf surface.

Specific leaf area (SLA) was determined for five plants per treatment from their fifth youngest leaf (counted from the shoot apex). Leaves were weighed to obtain fresh mass (FW), and their surface area estimated from a leaf scan using ImageJ software [93]. Leaves were then dried at 70 °C for 2 days and weighed again to determine dry mass (DM). SLA was calculated as the ratio of leaf area to leaf DM. Leaf water content (WC) was calculated as WC = [(FW-DM)/FW].

Sugar concentration was determined for photosynthetically active leaves from three plants per treatment. For sugar extraction, 0.8 g of frozen leaves was ground to a fine powder in liquid nitrogen, and free soluble sugars were extracted with 7 mL of 70% ethanol. The extracts were then centrifuged for 10 min at 8000× *g* at 15 °C. Total soluble sugars in the supernatant were quantified spectrophotometrically using the anthrone reagent method according to Yemm and Willis [94]; the resulting sugar concentration was expressed as milligrams sugars per gram of leaf FW.

### 4.3. Measurements of Floral Traits and Resources

The number of open flowers per plant was counted for five plants per treatment. For five flowers per treatment, corolla depth was measured as the length of the lower sepal.

Nectar was extracted with 10 μL glass capillary tubes (Hirschmann Laborgeräte, Eberstadt, Germany) from five flowers per treatment. Nectar extractions were performed on flowers at the same developmental stage in the afternoon in order to collect the daily nectar production. Total sugar concentration of nectar (C, g sucrose/100 g solution) was measured with a low-volume hand refractometer (Eclipse handheld refractometer; Bellingham and Stanley, Tunbridge Wells, UK).

The anther brushes were collected from ten randomly selected flower buds per treatment, 1 day before anthesis, and stored in FAA solution (70% ethanol, glacial acid acetic, 35% formaldehyde; 18:1:1). To count the number of pollen grains, each brush was crushed separately and placed in microfuge tubes containing 50 μL Alexander stain [95]. Tubes were then vortexed to disperse pollen grains in the solution. A subsample of 1 μL was used to count pollen grains on a microscope slide under a light microscope (Nikon Eclipse E400, G 400×). Counts were performed in triplicate for each stylar brush. Polypeptide content (molecular weight >10 kDa) of pollen was determined from 5 mg of dry pollen in triplicate for each treatment following the method described in Vanderplanck et al. [96]. Total polypeptides were quantified using the bicinchoninic acid (BCA) Protein Assay Kit (Pierce, Thermo Scientific), with bovine serum albumin (BSA) as standard.

### 4.4. Statistical Analyses

Normality of the data was estimated using QQ plots. Linear mixed models and analysis of variance (type II) were performed to a significance level of *p* < 0.05 to evaluate the effects of temperature increase, water stress and their interaction. For repeated measurements on the same plant at one time point (chlorophyll concentration, polypeptide concentration), linear mixed models were used with two fixed factors and their interaction (temperature × water) and plants as the repeated factor. Tukey’s HSD test was performed for post hoc analyses. To obtain a global view of the influence of rising temperature and water stress on all parameters, a principal component analysis (PCA) was performed. All analyses were performed in R 3.6.1 [97], using the packages *car* for F test, *lme4* for linear mixed models, *FactomineR* for PCA, *ggplot2* and *yarr* for plots. Data are presented as means ± standard errors (SE).

## Figures and Tables

**Table 1:** Statistical results of the effects of an increase in temperature (Temp), water stress (Water) and their interaction (Temp * Water) on vegetative, physiological and floral traits and resources of *Impatiens glandulifera.* Significant differences are indicated in bold.

Parameter	Temp	Water	Temp * Water
Number of leaves	F_2, 28_ = 1.66, *p* = 0.21	F_1, 28_ = 4.68, ***p* = 0.04**	F_2, 28_ = 0.24, *p* = 0.79
Specific leaf area	F_2, 24_ = 5.05, ***p* = 0.01**	F_1, 24_ = 2.15, *p* = 0.16	F_2, 24_ = 1.39, *p* = 0.27
Chlorophyll content	F_2, 22_ = 1.42, *p* = 0.16	F_1, 22_ = 0.05, *p* = 0.83	F_2, 20_ = 0.25, *p* = 0.78
Photosystem II efficiency	F_2, 23_ = 2.17, *p* = 0.14	F_1, 23_ = 11.94, ***p* = 0.002**	F_2, 23_ = 0.69, *p* = 0.51
Photochemical quenching	F_2, 23_ = 0.06, *p* = 0.95	F_1, 23_ = 14.14, ***p* = 0.001**	F_2, 23_ = 0.22, *p* = 0.80
Leaf sugar concentration	F_2, 12_ = 22.31, ***p <* 0.001**	F_1, 12_ = 7.99, ***p* = 0.02**	F_2, 12_ = 1.31, *p* = 0.31
Stomatal conductance	F_2, 19_ = 20.08, ***p <* 0.001**	F_1, 19_ = 2.51, *p* = 0.13	F_2, 19_ = 0.67, *p* = 0.67
Leaf water content	F_2, 24_ = 2.97, *p* = 0.07	F_1, 24_ = 1.22, *p* = 0.28	F_2, 24_ = 1.06, *p* = 0.36
Number of flowers	F_2, 28_ = 1.08, *p* = 0.35	F_1, 28_ = 14.98, ***p <* 0.001**	F_2, 28_ = 3.16, *p* = 0.06
Anthesis duration	F_2, 12_ = 22.29, ***p <* 0.001**	F_1, 12_ = 0.14, *p* = 0.71	F_2, 12_ = 0.57, *p* = 0.58
Corolla width	F_2, 24_ = 3.27, *p =* 0.06	F_1, 24_ = 4.85, ***p* = 0.04**	F_2, 24_ = 0.42, *p* = 0.66
Corolla depth	F_2, 24_ = 10.54, ***p******<*** **0.001**	F_1, 24_ = 0.28, *p* = 0.60	F_2, 24_ = 0.21, *p* = 0.82
Nectar volume	F_2, 19_ = 33.02, ***p <* 0.001**	F_1, 19_ = 23.79, ***p <* 0.001**	F_2, 19_ = 3.51, *p* = 0.05
Nectar sugar concentration	F_2, 19_ = 3.63, ***p* = 0.04**	F_1, 19_ = 0.10, *p* = 0.75	F_2, 19_ = 2.96, *p* = 0.08
Number of pollen grains ^1^	F_2, 36_ = 28.41, ***p <* 0.001**	F_1, 36_ = 1.37, *p* = 0.25	F_2, 36_ = 1.42, *p* = 0.24
Polypeptide concentration	F_2, 16_ = 11.01, ***p <* 0.001**	F_1, 66_ = 2.33, *p* = 0.15	F_2, 16_ = 21.07, *p* = 0.84

^1^ without 27WW, 27WS.

## Data Availability

The data presented in this study are available in the text. The data presented in this study are available on request from the corresponding author.
